# Unintentional ingestion of putative delta-8 tetrahydrocannabinol by two youth requiring critical care: a case report

**DOI:** 10.1186/s42238-023-00176-x

**Published:** 2023-03-21

**Authors:** Erin K. Bradley, Brooke E. Hoots, Evan S. Bradley, Douglas R. Roehler

**Affiliations:** 1 Department of Pediatric Critical Care Medicine, Children’s Healthcare of Atlanta, Atlanta, GA USA; 2 Cannabis Strategy Unit, Division of Overdose Prevention, National Center for Injury Prevention and Control, Centers for Disease Control and Prevention, 4770 Buford Hwy, NE, Atlanta, GA 30341 USA; 3 Department of Emergency Medicine, Division of Medical Toxicology, UMass Memorial Medical Center, University of Massachusetts Medical School, Worcester, MA USA

**Keywords:** Cannabis, Overdose, Pediatrics

## Abstract

**Background:**

Delta-8 tetrahydrocannabinol (THC) is a psychoactive cannabinoid from the cannabis plant that can be synthetically converted from cannabidiol (CBD). Most states permit the full or restricted sale of hemp and hemp-derived CBD products, and therefore, delta-8 THC products are on the rise. Delta-8 THC consumption can cause intoxication. Products are often sold in edible form and occasionally in packaging that appears similar to candy. Clinical presentations for delta-8 THC ingestions are understudied and may differ from those described for delta-9 THC ingestions.

**Case presentation:**

This case report describes unintentional ingestions of putative delta-8 THC by two pediatric patients that results in admission to the pediatric intensive care unit. The ingestions were of putative delta-8 THC infused product that resembled popular candies. Both patients developed periods of bradypnea with continued intermittent periods of agitation. Medical intervention included observation, noninvasive positive pressure ventilation via high flow nasal cannula, and intubation—but was not needed for both patients. Although family noted ongoing irritability for the patients, both were discharged approximately 45 h after ingestion. Delta-8 THC ingestion is reliant on self-report.

**Conclusions:**

As the availability of delta-8 THC increases, along with associated pediatric exposures, it is imperative for health care providers to quickly recognize and provide adequate treatment. While there is no specific antidote for THC intoxication beyond supportive care, providers can play an important role in prevention by educating parents and guardians on safe cannabis storage and by documenting cases for adverse event monitoring.

## Background

Delta-8 tetrahydrocannabinol (THC) is one of several psychoactive THC isomers in the cannabis plant, although most of cannabis’s psychoactive effect is ascribed to delta-9 THC (Hollister and Gillespie 1973). Marijuana refers to all parts of the cannabis plant containing > 0.3% delta-9 THC by dry weight, while hemp is any part of the cannabis plant containing ≤ 0.3% delta-9 THC by dry weight (U.S. Congress 2018). Most states permit the full or restricted sale of hemp and hemp-derived cannabidiol (CBD) products. Although CBD is a non-psychoactive cannabinoid of the cannabis plant, CBD can be synthetically converted into psychoactive delta-8 THC to produce larger amounts of delta-8 THC than found naturally in the cannabis plant (Rosenberg et al. 2015; Kiselak et al. 2020). Additionally, delta-8 THC products may be labeled as hemp or CBD, which consumers may not associate with psychoactive ingredients due to their derivation from CBD products (Centers for Disease Control and Prevention 2021). Delta-8 THC products are increasingly appearing in both marijuana and hemp marketplaces, some of which operate legally under state laws (Leas 2021). In May 2022, a federal appeals court issued a ruling that delta-8 THC is not a schedule 1 substance under federal law (AK Futures LLC v. Boyd St. Distro, LLC, 35 F.4th 682 (9th Cir. 2022)). Until the federal government clarifies its position, the regulation of delta-8 THC falls under the purview of the states, with many states banning or restricting sale of these products (Public Health Law Center at Mitchell Hamline School of Law 2022). Some delta-8 THC edible products are marketed to mimic popular candy brands (WSAZ News 2022; U.S. Food and Drug Administration 2022).

Several studies have documented increases in unintentional youth cannabis exposures following expansion of cannabis legalization over the past decade (Roehler et al. 2022; Tweet et al. 2023), with edibles being the most frequent route of exposure (Whitehill et al. 2021). With increased availability of delta-8 THC (Centers for Disease Control and Prevention 2021), including in states that have not passed laws to allow medical and/or non-medical adult cannabis use, there is concern that unintentional youth ingestions could continue to increase (U.S. Food and Drug Administration 2021). Clinical presentations for delta-8 THC ingestions are understudied and may differ from those described for delta-9 THC ingestions (Hollister and Gillespie 1973). The objective of this case report is to characterize the signs and symptoms of putative delta-8 THC youth ingestions, examples of treatment regimens for youth exposed to delta-8 THC, and preventative measures for unintentional cannabis poisoning.

### Case presentation

Two otherwise healthy sisters, ages 2 (A.R.) and 4 (T.R.), presented to an emergency department (ED) in a Southeastern state where neither medical nor nonmedical cannabis use is legal following unintentional ingestion of putative delta-8 THC candies. A.R. reportedly ingested a rope of candy designed to resemble a popular candy brand labeled as containing 500 mg (38 mg/kg) of delta-8 THC. T.R. ingested 7 out of 10 pieces of candy totaling about 350 mg (16 mg/kg) of delta-8 THC. Ingestion occurred at 9:15 PM. The parents reported that products labeled as containing delta-8 THC were acquired from a vape shop. Open and empty delta-8 THC-labeled packaging was found in the sisters’ hands, and no other THC-containing products were reported in the home.

On ED arrival approximately 1 h following ingestion, A.R. was crying and agitated. Vital signs (Table 1) were notable for mild tachycardia, but otherwise normal for age. On exam, A.R. had intermittent spells of agitation, moving hands and arms, and grasping at objects. The physical exam was otherwise unremarkable. Labs on admission were notable for urine drug screen positive for THC and negative for amphetamines, barbiturates, benzothiazines, cocaine, methadone, and opiates. Other screening labs, including complete metabolic panel (CMP) and complete blood count (CBC), were within normal limits. Acetaminophen, salicylate, and alcohol (ETOH) levels were undetectable. An electrocardiogram (EKG) demonstrated normal sinus rhythm (Table 1). At 12:00 AM, 3 h post ingestion, A.R. developed periods of bradypnea with continued intermittent periods of agitation. Her respiratory rate decreased to 6–9 breaths per minute. A.R. was placed on noninvasive positive pressure ventilation via high flow nasal cannula but continued to have intermittent apneic episodes and worsening lethargy. Glasgow coma scale was estimated to be 8. Venous blood gas demonstrated mild hypoventilation with a pH of 7.31 and partial pressure of carbon dioxide of 49. Due to declining mental status, she was intubated around 2:00 AM for airway protection with midazolam and rocuronium. A.R. was started on dexmedetomidine and fentanyl following intubation per ED sedation protocol and was admitted to the pediatric intensive care unit (PICU).

**Table 1:** Pertinent laboratory values for two pediatric critical care patients admitted for unintentional delta-8 THC ingestions

	A.R.^a^	T.R.^a^	Reference Value
Qualitative Urine Drug Screen	Positive THC	Positive THC	Negative
Acetaminophen (ug/mL)	< 2.0	< 2.0	10.0–30.0
Salicylates (mg/dL)	< 1.7	< 1.7	10.0–30.0
Alcohol (mg/dL)	< 3	< 3	< 10
THC metabolites, urine quantitative 11-Nor-9-carboxy-THC (ng/ml)	N/A	> 500	< 15
Vitals on admission			
Temperature (C)	36.6	36.1	36.5–37.5
Heart Rate (beats/min)	131	110	Tod 98–140; Pre 80–120
Respiratory rate (breath/min)	26	22	Tod 22–37; Pre 20–28
Blood pressure (mmHg)	119/83	99/63	Tod SPB 86–106/DBP 42–63 Pre 89–112/46–72
Electrolytes			
Sodium (mmol/L)	138	138	134–143
Potassium (mmol/L)	3.4	4.2	3.4–5.1
Chloride (mmol/L)	107	108	98–108
Bicarbonate (mmol/L)	24	26	19–29
Glucose (mg/dL)	108	91	70–110
BUN (mg/dL)	21	19	5–22
Creatinine (mg/dL)	.38	0.46	0.2–0.6
Liver function			
AST (u/L)	31	27	22–55
ALT (u/L)	24	20	11–30
CBC			
WBC (thou/uL)	12.18	8.79	5.0–15.5
Hemoglobin (g/dL)	11.3	10.6	11.5–13.5
Hematocrit (%)	35.1	33.6	34–40
Platelets (thou/uL)	279	269	150–450
Venous Blood Gas			
pH	7.31		7.32–7.42
pCO2 (mmHg)	49		38–50
pO2 (mmHg)	99		N/A
HCO3 (mmol/L)	25		22–28
Base Deficit	-1		-3–0
Capillary Blood Gas			
pH		7.33	7.35–7.45
pCO2 (mmHg)		44	35–45
pO2 (mmHg)		95	N/A
HCO3 (mmol)		25	20–28
Base Deficit		-2	-3–0
EKG	Normal	Normal	

*Abbreviations*: *ALT* Alanine Aminotransferase, *AST* Aspartate Aminotransferase, *BUN* Blood urea nitrogen, *CBC* Complete blood count, *DBP* Diastolic blood pressure, *EKG* Electrocardiogram, *Pre* Preschooler, *SBP* Systolic blood pressure, *Tod* Toddler, *THC* Tetrahydrocannabinol, *WBC* While blood cell

^a^A.R. and T.R. are the initials of the two pediatric patients. The patients’ initials were changed to ensure anonymity

Sedation drips were discontinued shortly after PICU arrival, around 8:00 AM. Pressure support trials were initiated; however, A.R. had continued apnea. A.R. remained off sedation and was successfully extubated at 7:00PM, almost 24 h after ingestion. A.R. remained somnolent overnight with bradypnea but remained stable on room air. Mental status improved the following morning; however, A.R. remained irritable. A.R. was transferred out of the PICU and discharged later that day around 6:00 PM, 45 h after ingestion.

On arrival to the ED, T.R. appeared somnolent but was arousable to stimulation. Her physical exam was otherwise unremarkable. Vital signs were unremarkable, and urine toxicology screen was positive for THC. CMP and CBC were normal. Acetaminophen, salicylate, and ETOH levels were undetectable (Table 1). EKG was normal sinus rhythm. At around 12:30 AM, T.R. became bradypneic and only arousable to sternal rub. Glasgow coma scale at that time was 8. Capillary blood gas was obtained and showed adequate ventilation. T.R. was placed on high flow nasal cannula (HFNC) at 8 L per minute. Her respiratory rate improved from 7 to 12 to 11–12 breaths per minute. T.R. was admitted to PICU.

On PICU arrival, T.R. was arousable with noxious stimuli and continued to maintain her airway. Her respiratory pattern improved, and she became easily arousable. HFNC was removed at 9:00 AM, 12 h after ingestion. She remained in PICU due to continued somnolence. The following morning, T.R. was more awake and able to eat by mouth. Although family noted ongoing irritability, T.R. was transferred out of the PICU and discharged later that day, about 45 h after ingestion. T.R.’s confirmatory lab result for quantitative urine THC returned post discharge was > 500 ng/mL. Initials of the patients have been changed for anonymity.

## Discussion

As edible products containing delta-9 THC have become more commonplace after increasing legalization of medical and/or nonmedical cannabis use, cases of unintentional delta-9 THC ingestion in children under 6 have risen (Boadu et al. 2020). The two current cases illustrate that the most serious manifestations of delta-9 THC exposure in children can also be observed in children exposed to suspected delta-8 THC. The rise in delta-8 THC products has increased the availability of psychoactive cannabis products, even in states where medical and/or non-medical adult cannabis use is not permitted under law (Centers for Disease Control and Prevention 2021). One social media analysis indicated that conversations about delta-8 THC increased 163% from December 2020 to April 2021 (Brightfield Group 2021). The analysis also demonstrated that delta-8 THC consumers were more likely to live in states with restrictive or no cannabis legalization. Thus, pediatric cannabis exposures may become more common due to the wide availability of delta-8 THC products, especially in states that have not legalized adult nonmedical cannabis use and lack standards for product labeling (Brightfield Group 2021). Improving clinician awareness that pediatric exposures to this compound carry similar risk to delta-9 THC exposure could help guide treatment decisions.

Currently, data are limited on pediatric delta-8 THC ingestions. The clinical effects and pharmacology of delta-8 THC are thought to be similar to delta-9 THC due to their similar molecular structures (Babalonis et al. 2021; Wurz et al. 2022). Although there are few studies of delta-8 THC exposure, our findings demonstrate that clinical effects could reasonably mimic delta-9 THC in children. Signs and symptoms of pediatric delta-9 THC ingestion most often include lethargy, ataxia, mydriasis, tachycardia, and hypotonia (Richards et al. 2017). Commonly available and standard urine drug screens detect a delta-9 THC metabolite that peaks in the urine 3–5 days after ingestion and cannot be used to diagnose acute intoxication, although they can later be used to confirm exposure (Blohm et al. 2019). Major urinary metabolites of delta-8 THC and delta-9 THC differ only by the placement of one double bond and have similar molecular weights. Therefore, there is high antibody cross-reactivity in the immunoassay between delta-8 and delta-9 THC. Patients using delta-8 THC will likely screen positive for cannabinoids, either from delta-8 THC metabolites or from other cannabinoids in the product. Confirmatory testing done in hospitals is designed to identify delta-9 THC and metabolites, and hospital toxicology would not have confirmed the parental report of exposure to delta-8 THC (Cary 2021). While delta-8 THC confirmatory testing is possible through specific protocols utilizing liquid chromatography-quadrupole time-of-flight mass spectrometry at forensic centers of excellence (Akpunonu et al. 2021), this is not commonly ordered. This may be due to lack of awareness by the physician or lack of availability of testing from forensic labs that are contracted with most medical centers where testing for delta-8 THC is not commonplace. Standard confirmatory testing by gas chromatography/mass spectrometry or liquid chromatography with tandem mass spectrometry does not routinely search for delta-8 THC metabolites and confirmatory testing for delta-9 THC metabolites performed on these samples may yield indeterminate results (Chan-Hosokawa 2021). A patient may therefore have a positive urine cannabinoid screening test and a negative confirmatory test. This can lead to diagnostic uncertainty if the history of exposure to delta-8 THC is not available and may lead to unnecessary additional testing for altered mental status in pediatric patients. In the current cases, confirmatory testing that distinguished between delta-8 THC and delta-9 THC was not completed, and identification of delta-8 THC in these cases is reliant on self-report. Although delta-8 THC confirmatory testing is the gold standard for the current case report, and while widespread delta-8 THC confirmatory testing would help confirm exposures in situations where history is unclear and may reveal unique differences in presentation and sequelae between delta-8 and delta-9 THC cases, such testing availability is not widely available and providers often must rely on patient self-report. From a clinical practice perspective, the pharmacology of delta-8 THC is similar to delta-9 THC in humans, meaning that it has similar metabolism. However, it does produce a different balance of cannabinoid receptor activation and a different rate of metabolite formation. The clinical dose-response and concentration-response of delta-8 THC has not been studied relative to human pharmacokinetics (Tagen and Klumpers 2022). This would suggest that delta-8 THC ingestions and delta-9 THC ingestions would require similar monitoring (especially as delta-8 products may also contain high amounts of delta-9 THC). However, due to the unregulated nature of delta-8 THC products, there may be a higher content ingested than intended, and patients may need a longer duration of monitoring. In addition, due to the proliferation of these products in states without legal cannabis marketplaces, many of these products may not be tested systematically for contaminants such as heavy metals, solvents, or pesticides that may have adverse health effects. This case report reflects the reality of an absence of confirmatory delta-8 THC testing for many health care settings. In September 2021, the Centers for Disease Control and Prevention (CDC) released a health advisory through their Health Alert Network (HAN) on the increase in delta-8 THC cannabis products and associated adverse events (Centers for Disease Control and Prevention 2021). CDC alerted clinicians that insufficient labeling of the psychoactive effect of delta-8 THC products, their availability in states that have not legalized nonmedical adult cannabis use, and availability outside regulated dispensaries in states with legal adult cannabis use could result in unexpected THC intoxication. Syndromic surveillance of delta-8 THC-associated ED visits showed that ED visits were more common in regions of the USA where states have not legalized nonmedical adult cannabis use. Confirmatory testing for delta-8 THC among these exposures, especially among those who become critically ill as are described in this case report, would lead to a better understanding of the impacts of delta-8 THC on public health and could help direct policy measures to mitigate exposures.

Delta-8 THC products may be labeled as “hemp,” which could mislead consumers who associate hemp with non-psychoactive products (U.S. Food and Drug Administration 2021). In addition, delta-8 THC products are not systematically tested for contaminants that may be introduced during the CBD conversion process that may have adverse health effects (Delta-8-THC 2022). A recent study analyzed major components of 27 products from 10 e-cigarette brands containing delta-8 THC (Meehan-Atrash and Rahman 2021). None of the products had accurate delta-8 THC concentration labeled on the packaging and all contained reaction side-products, including heavy metals and delta-9 THC.

To prevent exposures, providers can educate parents and guardians on how to ensure that cannabis products are locked, hidden, and secured using child-resistant packing to limit youth access. In a recent survey of adults in a pediatric ED, only 45% of people who used cannabis at home reported that their cannabis was locked and hidden, demonstrating the opportunity for provider education on safe storage (Gimelli et al. 2021). Policymakers in states that allow for nonmedical adult cannabis use may consider education campaigns or product warnings on safe cannabis storage, as well as stricter restrictions on product packaging and labeling that appeal to children.

## Conclusion

Given increases in pediatric cannabis exposures and delta-8 THC product availability, it is important for providers to be aware of pediatric presentations following unintentional THC ingestion. Also, given the emergence of additional hemp-derived psychoactive cannabinoids into the marketplace (e.g., THC-O acetate, delta-10 THC, hexahydrocannabinol [HHC]), surveillance of adverse events associated with these products is increasingly important. While there is no specific antidote for THC intoxication beyond supportive care, providers can play an important role in prevention by educating parents and guardians on safe cannabis storage, and by documenting cases for adverse event monitoring. As cases increase, expanding policies related to product labeling and packaging may help protect youth from unintentional cannabis ingestions.

## Data Availability

All data analyzed during this study are included in this published article.
